# Potential of Wood Processing Residues as Eco-Friendly Adsorbents for Wastewater Treatment

**DOI:** 10.3390/ma19030578

**Published:** 2026-02-02

**Authors:** Silviya Lavrova, Nikolay Yavorov

**Affiliations:** 1 Department of Environmental Engineering, University of Chemical Technology and Metallurgy, 1797 Sofia, Bulgaria; 2 Department of Pulp, Paper and Printing Arts, University of Chemical Technology and Metallurgy, 1797 Sofia, Bulgaria; nyavorof@uctm.edu

**Keywords:** biomass utilization, wood shavings, adsorption, manganese, water treatment

## Abstract

In the context of global warming mitigation through energy conservation and pollution control, integrating green waste into treatment processes has become more popular. This study evaluated the potential of raw wood processing residues generated from furniture manufacturing as renewable sorbents for water treatment. Comparative studies assessed the Mn(II) removal efficiency of raw walnut (WW) and cherry (CW) wood shavings and the derived biochars (BChWW, BChCW) produced by hydropyrolysis. SEM, BET, FTIR, and TGA analyses characterized their surface and structural properties. CW demonstrated a higher adsorption capacity compared to WW. Physical activation enhanced the surface properties and Mn(II) adsorption affinity of the materials. Maximum adsorption capacities ranged from 2.1 to 2.2 mg/g for CW and WW, and 2.4 to 2.5 mg/g for BChCW and BChWW. The Freundlich model best fits to the data obtained using CW (R^2^ = 0.997) and BChCW (R^2^ = 0.984), while the RALF isotherm almost perfectly describes the mechanism of the Mn(II) adsorption onto WW (R^2^ = 0.999) and BChWW (R^2^ = 1.000). The pseudo second-order kinetic model shows strong agreement with experimental data, which suggests chemisorption on a heterogeneous surface. The results underscore the potential of wood industry byproducts as efficient and low-cost adsorbents for water treatment, supporting the circular economy and sustainable environmental management.

## 1. Introduction

The increasing presence of potentially toxic elements (PTEs) in the aquatic ecosystems represents a significant threat to the environment and public health. These elements, including essential and non-essential metals originating from both anthropogenic and natural sources, enter water basins, where they persist and bioaccumulate even at trace concentrations, disrupting physiological and biochemical processes in the living organisms [1,2].

Manganese (Mn) is a metal that is part of the PTEs and could be found as an essential micronutrient or as a potential contaminant. In living organisms, it is required at physiological concentrations for the normal enzymatic function and metabolic activity. However, the excessed manganese permissible limits or chronic exposure to its influence, induce neurotoxic effects, including cognitive impairment, behavioral disorders, and Parkinson-like symptoms also [3,4,5,6]. Industrial activities, such as mining, ferroalloy production, battery manufacturing, textile dyeing, as well as wastewater discharge from tanneries and chemical plants, are primary anthropogenic sources of manganese contamination in surface and groundwater systems [7,8]. In addition, the erosion of the manganese-rich rocks contributes to environmental loading, particularly under conditions that enhance the manganese solubility and mobility [9].

Various technologies have been developed for metal contamination removal from wastewater, including chemical precipitation, ion exchange, adsorption, and other [2,10]. However, many of these methods are cost-intensive, energy-consuming, or generate secondary pollutants. In contrast, adsorption is considered a simple, effective, and financially profitable method, proper for decentralized water treatment systems. In contrast, adsorption is considered a simple, effective, and financially profitable method, proper for decentralized water treatment systems.

Activated carbon is one of the most used adsorbents due to its high specific surface area and strong affinity for metal ions. However, its production costs are relatively high, and the complexity of regeneration processes limits its large-scale application [11]. That is why there is growing attention to the development of low-cost, sustainable adsorbents derived from natural biomass, agricultural residues, and industrial byproducts.

Lignocellulosic materials, like as sawdust [12,13], wood barks [14], wood shavings [15], fruit shells [16,17], and others, attract attention as a potential low-cost adsorbents, especially for metal ions removal. On their surfaces, these raw materials have functional groups, such as hydroxyl, carboxyl, and phenolic moieties, which directly bind metal ions [18,19]. Although the adsorption performance of such materials is generally lower than that of their chemically or thermally modified analogs, some authors report that the raw biomass can achieve approximately 50% removal of manganese from aqueous media under optimized conditions [16,20].

To enhance adsorption efficiency, chemical modification of lignocellulosic biomass through acid or base treatment, oxidation, or functionalization with complexation agents has been widely investigated. As a result of such modifications, an increase in the active sites binding density, and change surface charge characteristics, resulting in improved metal uptake [14,21]. In parallel, thermal conversion of biomass into biochar or activated carbon via pyrolysis and activation processes produces carbonaceous materials with tailored porosity and surface chemistry, further enhancing their adsorption performance toward metal ions [22].

Sustainable wastewater treatment encourages the use of low-cost, biomass-derived adsorbents, which are renewable and eco-friendly alternatives to conventional chemical-based methods [23]. The vast amounts of lignocellulosic residues from large-scale industrial sectors, such as paper production, have attracted scientific attention for a long time due to their favorable structure, abundance of functional groups, and wide availability. However, a significant amount of wood-based by-products with high lignin content, structural stability, and accessibility generated by the furniture and woodworking industries remain underutilized. These residues are promising materials for cost-effective, sustainable removal of metal contaminants from aquatic environments.

This study aims to evaluate the potential of raw wood processing residues from walnut (*Juglans regia*) and cherry (*Prunus avium*) furniture manufacturing, along with their corresponding biochars, as efficient, low-cost, and renewable adsorbents for the removal of manganese ions from mining wastewater. In contrast to previous studies predominantly focused on chemically modified biosorbents or nut shells, this work investigates unmodified wood residues and their biochars obtained at 700 °C. The study provides a novel insight into the valorization of industrial lignocellulosic by-products within the circular economy framework and contributes new experimental data on Mn(II) adsorption using these underexplored materials.

## 2. Materials and Methods

### 2.1. Materials

The investigations were performed using waste materials from industrial wood processing. The feedstock consisted of shavings generated during the planing of furniture components manufactured from cherry *(Prunus avium* L.) and walnut (*Juglans regia* L.). These residues were obtained from Forest Parquet International Ltd., located in Gabarevo, Bulgaria (42°37′58.7″ N, 25°09′52.3″ E). The collected samples were air-dried to reduce surface moisture and stored in sealed polyethylene containers prior to further processing and analysis.

The reagents used in the experimental and analytical work are of analytical grade.

### 2.2. Preparation of Adsorbent

To evaluate the adsorption potential of raw wood processing residues from walnut (WW) and cherry (CW) for water treatment applications, the raw materials were physically modified, i.e., experimental work was carried out with two types of adsorbents—raw and physically modified wood processing residues from walnut (BChWW) and cherry (BChCW).

The physical activation via controlled hydropyrolysis in a laboratory-scale vertical tubular furnace reactor under a continuous nitrogen flow to maintain an inert atmosphere. The system, operated according to principles detailed in Patent BG63594B1 [24], employed a gradual temperature increase up to 700 °C at a heating rate of approximately 10 °C min^−1^. The samples were held at the final temperature for 60 min under atmospheric pressure, with a constant N_2_ flow rate of 100 mL min^−1^ to ensure uniform heat distribution and prevent oxidation. The biomass-to-reactor volume ratio was maintained at approximately 1:10 (*w*/*v*), corresponding to a reactor loading of about 10% of its total volume. Under these thermal conditions, simultaneous carbonization and in situ activation occurred, yielding biochar with enhanced surface area and structural stability.

### 2.3. Characterization of Adsorbents

The wood processing residues and their corresponding biochar products were subjected to comprehensive physicochemical characterization prior to their use in adsorption studies. In addition, adsorption capacity tests were conducted to evaluate their suitability for wastewater treatment applications.

To evaluate the potential of the raw residues, a detailed analysis of their chemical composition was carried out. The residues were initially milled to pass through a 0.6 mm sieve to ensure particle-size uniformity and facilitate complete interaction with reagents during subsequent chemical analyses. Before all determinations, the moisture content of the milled samples was measured in accordance with TAPPI T 264 cm-22 [25], allowing analytical results to be expressed on an oven-dry basis.

The extractives content was determined gravimetrically in accordance with TAPPI T 204 cm-17, after Soxhlet extraction of 10 g of biomass with 150 mL of an ethanol-benzene mixture (1:2, *v*/*v*) [26]. The hot-water solubility was determined according to the TAPPI T 207 cm-22 standard, which characterizes the fraction of low-molecular-weight compounds such as starches, tannins, gums, simple sugars, coloring substances, and inorganic salts [27].

The extractive-free samples were further analyzed for their lignin content according to TAPPI T 222 om-21 and cellulose content following the Kürschner–Hoffer method [28,29]. In addition, the ash content was determined in accordance with the TAPPI T 413 om-22 standard by complete combustion of the samples in a muffle furnace at 900 ± 25 °C until constant weight was achieved [30].

The chemical composition of the adsorbents was determined by Fourier transform infrared spectroscopy (Nicolet iS50 FTIR-ATR, Thermo Scientific, Waltham, MA, USA) in the wavenumber range of 400–4000 cm^−1^. Quantitative evaluation of the characteristic functional groups was performed by determining both the band intensities (minimum transmittance) and the integrated areas under the selected absorption bands. The integration of each band area was carried out numerically using the trapezoidal rule, implemented in MATLAB (version R2025b) via the OriginPro 9.1 MATLAB Console (OriginLab Corporation, Northampton, MA, USA). The numerical integration followed Equation (1):

(1) $$A=\sum_{i=1}^{n-1}\frac{\left(y_{i}+y_{i+1}\right)}{2}(x_{i+1}-x_{i}),$$

where *A* is the integrated band area, *y_i_* and *y_i_*_+1_ are the transmittance values, and *x_i_* and *x_i_*_+1_ are the corresponding wavenumbers (cm^−1^). The accuracy and reproducibility of the numerical integration were verified by repeating the calculations with a refined step size (Δx = 1 cm^−1^). The relative deviation between successive integrations did not exceed 2%, confirming the numerical stability of the applied method. All spectra were baseline-corrected and normalized before integration to ensure consistent comparison among samples.

The morphological structure of the adsorbent’s surface was analyzed using a field-emission scanning electron microscope (SEM, EVO 10, Carl Zeiss GmbH, Oberkochen, Germany) equipped with an energy-dispersive spectrometer (EDS, Oxford Instruments, Xplore, High Wycombe, UK). Thermogravimetric analyses were performed using a thermal analyzer (NETZSCH STA 449F3, Netzsch Gerätebau GmbH, Selb, Germany) at a heating rate of 10 K.min^−1^, from 20 °C to 900 °C under an Ar atmosphere. The specific surface area, total volume, and pore size of the adsorbents were obtained by Brunauer–Emmett–Teller Surface Area Analysis (BET) using NOVAtouch 4LX (Quantachrome instruments, Boynton Beach, FL, USA) after prior degassing at 150 °C for 240 min under vacuum. The metal ion concentration was determined by Inductively Coupled Plasma-Optical Emission Spectroscopy (“Prodigy” High dispersion ICP-OES, Teledyne Leeman Labs, Hudson, NY, USA).

All analyses were performed in triplicate, and the results are expressed as percent of oven-dry mass to ensure precision, accuracy, and comparability among measurements. This comprehensive characterization enabled a reliable assessment of the main chemical constituents, which is essential for evaluating the suitability of the residues for subsequent utilization, particularly for biochar production through pyrolysis.

### 2.4. Determination of the Adsorption Efficiency of Adsorbents

The raw wood processing residues were initially pre-washed with distilled water to eliminate impurities, followed by drying at room temperature until a constant weight was achieved. Subsequently, a series of experiments were conducted using these green adsorbents and their biochars.

The study investigates the adsorption behavior of raw wood processing residues and their derived biochars in a single-component aqueous medium containing manganese ions. The Mn(II) stock solution (100 mg/L) was prepared by dissolving 0.3077 g MnSO_4_·H_2_O in 1000 mL of distilled water. Then, the stock solution was diluted to prepare experimental solutions of the desired concentration.

Batch experiments were conducted in 100 mL conical flasks containing 50 mL of Mn(II) solutions with concentrations ranging from 2 to 50 mg/L and 1 g adsorbent dosed using an analytical balance (AS 110/C/2, RADWAG, Radom, Poland). The suspensions were agitated at 200 rpm using a digital orbital shaker (SK-O330-Pro, DLAB Scientific Co., Shunyi, China) for 24 h at ambient temperature to ensure thorough mixing of the biosorbent with the aqueous medium containing manganese ions. After filtration through ‘blue ribbon’ filter paper, the residual concentration of manganese ions in the supernatant was determined using inductively coupled plasma optical emission spectrometry (ICP-OES). The measurements were repeated three times, and the average values were taken.

The adsorption kinetics of manganese ions was studied using an initial concentration of 15 mg/L, and time intervals of 3, 5, 10, 20, 40, and 60 min.

The equilibrium capacity (*q_e_*) of the adsorbents was calculated using Equation (2):

(2) $$q_{e}=\frac{(C_{0}-C_{e})V}{m},$$

where *C*_0_ is the initial metal ion concentration (mg/L), *C_e_* is the equilibrium metal ion concentration (mg/L), *V* is the solution volume (L), and m is the mass of the adsorbent (g).

The adsorption studies were performed at unmodified pH values ranging from 5.4 ± 0.17 to 5.8 ± 0.21, reflecting the pH values observed at actual copper mining sites. Similarly, the removal efficiency (*RE*, %) was calculated using Equation (3):

(3) $$RE=\frac{\left(C_{0}-C_{e}\right)}{C_{0}}100,$$

### 2.5. Equilibrium and Kinetic Modeling

To better understand the solid–liquid adsorption process of manganese ions on the different biomaterials used, the experimental data were applied to the Langmuir, Freundlich, Baudu, and RALF nonlinear isotherm models [31,32,33], and to three nonlinear kinetics models: pseudo-second order, Elovich, and intra-particle diffusion [34,35,36], using Microsoft Excel 365/XLSTAT Advanced (Lumivero, Denver, CO, USA, 2023, Addinsoft, Paris, France) at nonlinear regression with a convergence criterion of 0.000001, and a maximum of 1000 iterations. The models used are presented in Table 1. To identify the most representative adsorption model for each adsorbent, a combination of statistical and physical evaluation criteria was employed. Goodness of fit was primarily assessed using the coefficient of determination (R^2^), with values approaching unity indicating a high degree of model fit to the experimental data. In addition, error functions including the sum of squared errors (SSE), mean squared error (MSE), and root mean squared error (RMSE) were utilized to quantify the deviation between predicted and observed values, with lower values signifying superior predictive accuracy. Beyond statistical metrics, model selection also considered physical plausibility. Parameter realism was a key criterion, requiring all estimated constants to exhibit numerically sensible values such as nonnegative adsorption capacities and reasonable values, reflective of physically meaningful adsorption intensities. Furthermore, physical consistency was evaluated by examining whether model parameters showed trends consistent with known physicochemical properties of the materials, such as porosity, surface functional groups, and ash content. Only models that satisfied both statistical robustness and physical interpretability were deemed valid representations of the adsorption system.

## 3. Results

### 3.1. Characterization of Raw Wood Processing Residues and Their Biochar Products

A comprehensive characterization of the investigated wood processing residues was carried out to evaluate their potential as raw adsorbents and precursors for biochar production. As previously mentioned, the chemical composition of lignocellulosic biomass is of essential importance for its thermal behavior and strongly influences the properties of the resulting carbon material. The relative proportions of polysaccharides (cellulose and hemicelluloses), lignin, and extractives affect pyrolysis behavior and the characteristics of the final product. The biomass samples were analyzed using standardized methods to ensure accuracy and reproducibility.

The chemical composition of WW and CW residues highlights their suitability as lignocellulosic raw materials for green adsorbents and precursors in thermochemical valorization (Table 2).

The relatively high cellulose content in both biomasses (47–50%) suggests strong structural integrity and high potential carbon yield, while the moderate to high lignin content (18–21%) contributes to the aromaticity and chemical stability essential for surface functionality. Notably, walnut residues exhibited higher levels of lignin and ethanol-benzene extractables, indicating potential for adsorption of hydrophobic organic compounds due to increased surface affinity. In contrast, cherry residues displayed significantly lower ash content, which is advantageous for thermal conversion processes as it reduces inorganic interference during activation and improves pore development [17].

These compositional differences imply that each biomass offers distinct advantages. Cherry wood is particularly promising for thermochemical processing due to its low ash and high cellulose, while walnut wood, with richer aromatic fractions and bioactive extractives, could favor direct adsorption applications or functionalization routes post-activation. The moisture content of both residues (~13%) aligns well with the requirements of hydrothermal carbonization, a process that benefits from wet feedstock and enables in situ modification of carbon structure under mild conditions. The literature confirms that hydrothermal carbonization of walnut shells and other hardwood residues leads to hydrochars with high fixed carbon content and oxygen-rich surface functionalities, enhancing their affinity for heavy metals and organic pollutants in aqueous media.

**Figure 1:**
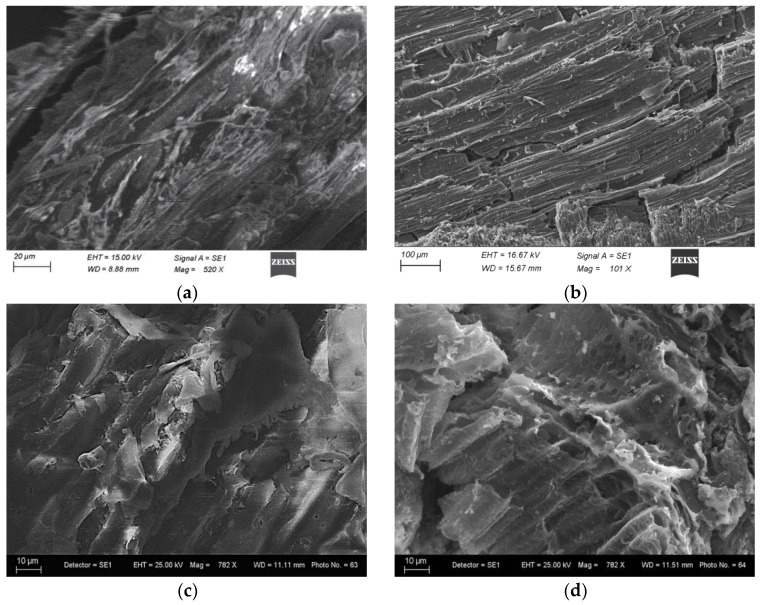
The SEM adsorbents surface morphology: (**a**) CW before activation; (**b**) BChCW; (**c**) WW; (**d**) BChWW.

SEM micrographs of CW, WW, BChCW, and BChWW (Figure 1) reveal that the untreated cherry and walnut wood processing residues possess relatively smooth, compact surfaces with limited visible porosity, a typical feature of unmodified lignocellulosic materials. Following physical activation via hydropyrolysis, a marked transformation in surface morphology is observed, characterized by increased roughness, the emergence of irregular pore structures, and the formation of numerous cavities and cracks. This physical modification induces significant structural transformation and promotes pore development because of volatilisation-driven degradation of the biomass matrix.

The structural transformation of the lignocellulosic matrix due to the pyrolysis was evident in both the surface morphology and the textural properties change in the materials studied.

**Figure 2:**
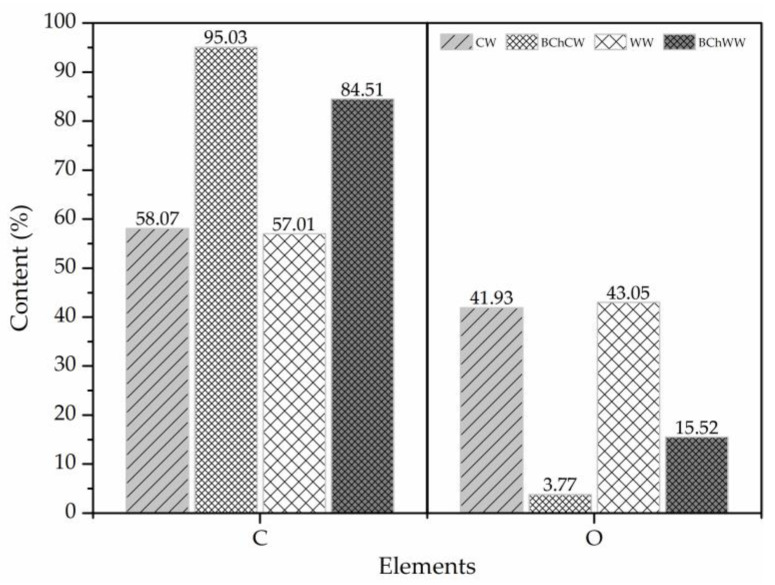
EDS of adsorbents.

Elemental analysis by energy-dispersive X-ray spectroscopy (EDS) revealed pronounced compositional changes induced by pyrolysis. Notably, the carbon content in CW increased substantially from 58.07% to 95.03% following thermal treatment, resulting in the formation of BChCW (Figure 2).

A similar trend was observed for WW, where pyrolysis elevated the carbon concentration from 57.01% to 84.51% in the derived BChWW. Concurrently, a significant reduction in oxygen content was detected, declining from 41.93% to 3.77% for CW and BChCW, and from 43.05% to 15.52% for WW and BChWW, respectively. These changes underscore the effective thermal decomposition of oxygenated functional groups, and the concurrent enrichment of carbonaceous structures, characteristic of highly porous biochar matrices formed during pyrolysis.

The BET results shown in Table 3 provide evidence of a significant increase in specific surface area and pore volume following pyrolysis. Raw samples exhibited very low surface areas and negligible pore volume, with pore width corresponding to mesoporous surfaces. Conversely, the biochars demonstrated well-developed porosity above 300 m^2^/g, with significant microporous existence with volumes near 0.3 cm^3^/g and pore widths around 1 nm. These results confirm the critical role of pyrolysis in developing accessible adsorptive sites.

**Figure 3:**
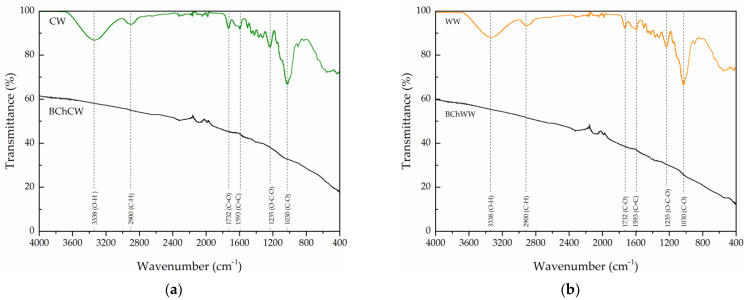
FTIR spectra of raw wood processing residues and their biochar products: (**a**) cherry wood (CW and BChCW) and (**b**) walnut wood (WW and BChWW).

The FTIR spectra of the studied adsorbents (Figure 3a,b) show the typical vibrational bands of lignocellulosic biomass, highlighting the structural transformations occurring during pyrolysis.

For CW and WW, a broad absorption band at 3338 cm^−1^ is assigned to O–H stretching in hydroxyl groups of cellulose and hemicelluloses. The band at 2900 cm^−1^ originates from aliphatic C–H stretching in methyl and methylene groups of polysaccharides. The distinct band around 1732 cm^−1^ corresponds to C=O stretching in carbonyl, carboxyl, and acetyl groups of hemicelluloses. A pronounced band at 1593 cm^−1^ is attributed to aromatic C=C stretching vibrations in lignin structures, consistent with previously reported FTIR profiles of wood biomass [37,38]. The region between 1600 and 1400 cm^−1^ is related to asymmetric C=C and C–C skeletal vibrations typical of lignin-derived aromatic structures [39]. The broad absorption band at approximately 1030 cm^−1^, accompanied by a less intense band at 1235 cm^−1^, can be attributed to multiple C–O and O–C–O stretching vibrations typical of carbohydrate-related structures [40].

In contrast, the FTIR spectra of both biochars (BChCW and BChWW) show the disappearance or significant attenuation of several major absorption bands observed in the raw samples (CW and WW), including those assigned to hydroxyl O–H stretching (3338 cm^−1^), aliphatic C–H stretching (2900 cm^−1^), and carbonyl C=O stretching (1732 cm^−1^). This reduction confirms that extensive dehydration, decarboxylation, and dehydroxylation reactions occurred during pyrolysis, leading to the removal of oxygenated functional groups and the formation of a carbon-enriched structure. The biochars show a weak band at 1593 cm^−1^ attributed to aromatic C=C stretching, along with additional bands in the 850–750 cm^−1^ region, corresponding to out-of-plane C–H deformations in aromatic rings [40,41]. These spectral features provide clear evidence for the development of condensed aromatic domains and progressive carbonization within the lignocellulosic matrix during the pyrolysis of CW and WW. The disappearance of the band near 1030 cm^−1^, corresponding to C–O–C and C–O stretching in polysaccharides, indicates the cleavage of ether and alcohol linkages [37].

These spectral changes confirm the progressive transformation of oxygenated functional groups into aromatic carbon domains with incipient graphitic order. This structural evolution enhances molecular organization and reduces surface polarity, thereby improving the biochars’ adsorption performance. Mechanistically, this spectral evolution reflects sequential dehydration, decarboxylation, and dehydroxylation of lignocellulosic components followed by condensation of lignin fragments into conjugated aromatic structures. This transformation marks the transition from oxygen-rich, polar biopolymers to structurally ordered sp^2^-hybridized carbon networks, demonstrating advanced carbonization and aromatic stabilization of the biochars.

**Figure 4:**
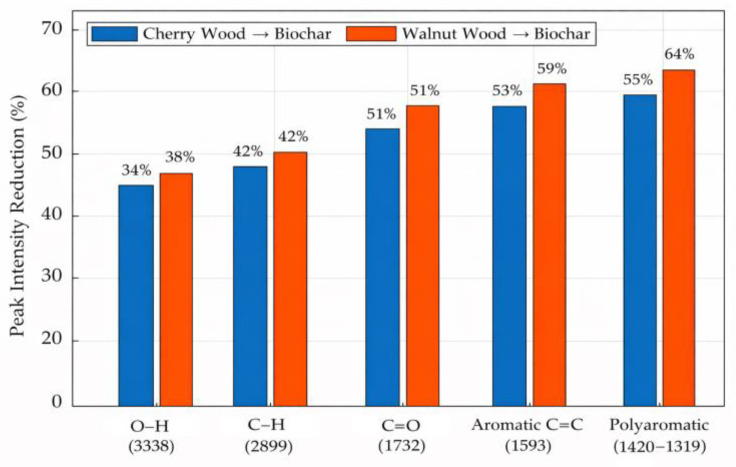
Quantitative FTIR analysis data of raw and carbonized samples.

Quantitative FTIR analysis (Figure 4) revealed a 34–46% decrease in the hydroxyl and aliphatic C–H bands and more than 50% reduction in the carbonyl (C=O) band, while the relative intensity of the aromatic and polyaromatic bands increased.

These spectral transformations confirmed the conversion of polar oxygen-containing moieties into stable aromatic carbon structures, enhancing π-π conjugation and structural ordering within the biochar matrix [39,40]. The observed evolution of functional groups suggests that thermal treatment plays a key role in developing carbonized structures characterized by lower surface polarity and enhanced aromatic stability.

**Figure 5:**
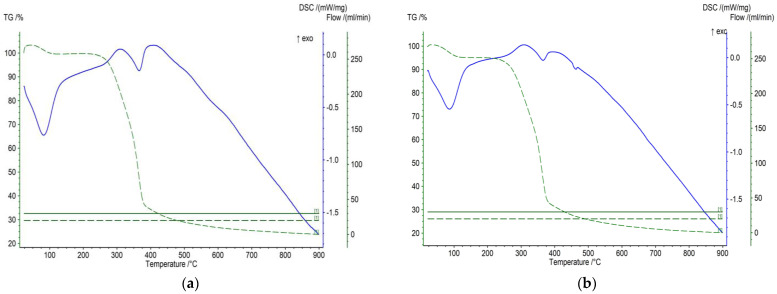
TGA and DSC curves of raw (**a**) cherry and (**b**) walnut wood shavings showing the main thermal degradation stages of the lignocellulosic matrix.

TGA/DTG analysis is an effective method for evaluating the content of hemicellulose, cellulose, and lignin using their characteristic thermal degradation profiles and for assessing compositional changes in biomass subjected to treatment [42,43]. The TGA analysis (green dashed line) shows the sample’s mass change (%) as a function of temperature. In contrast, the DSC curve (blue line) records endothermic and exothermic effects associated with the decomposition of the wood components. Cherry and walnut wood, like other types of lignocellulosic materials, mainly contain cellulose, hemicellulose, lignin, and moisture. Each of these components decomposes in a different temperature range, which determines the multi-stage nature of the process. The results shown in Figure 5, demonstrate an initial stage of moisture loss (~100 °C), major devolatilization between 200 and 380 °C, attributed to hemicellulose and cellulose decomposition during which the main weight loss is observed (~60–70%). The gradual decrease in the DSC signal above 400 °C is associated with the continued decomposition of lignin. The selected pyrolysis temperature of 700 °C for biochar production corresponds to the final stage of decomposition, ensuring complete carbonization of the precursors. Above this temperature, the so-called carbonization phase occurs, during which the reaction is practically complete, and a thermally stable carbon residue forms, accounting for about 25–30% of the initial mass.

All analyses demonstrate that raw wood processing residues (CW, WW) do not possess sufficiently good morphological and textural features compared with their resulting biochars, which exhibit high surface area, well-developed microporosity, stable aromatic structures, and minimal surface polarity due to the significant reduction of oxygenated surface groups, as confirmed by EDS. These biochar (BChCW, BChWW) characteristics, resulting from hydropyrolysis, are essential for efficient adsorbents. That means the thermal modification via pyrolysis is crucial in transforming agricultural residues into high-performance carbon-based adsorbents. These results support the strategic value of pyrolysis-based waste valorization for circular economy applications, especially in environmental remediation contexts, such as the removal of toxic pollutants from wastewater.

### 3.2. Determination of the Adsorption Efficiency of Raw Wood Processing Residues and Their Biochar Products

Adsorption experiments use Mn(II) concentrations ranging from 2 to 50 mg/L, selected to reflect realistic conditions observed in mine wastewater from operational copper mining sites in Bulgaria [44,45,46,47], where the pH and Mn(II) concentrations ranged from 3.8 to 5.5, and from 5 to 30 mg/L, respectively, with seasonal peaks reaching up to 50 mg/L. The other reasons for this concentration range selection stem from the widespread occurrence and persistence of Mn(II) in the environment, which pose health risks to humans and ecosystems, and from its limited removal efficiency under near-neutral pH conditions [44,45,46,47,48,49,50,51]. By reflecting realistic field conditions, the adopted experimental design provides a robust basis for evaluating the adsorption efficiency of the tested raw residues and their biochars.

**Figure 6:**
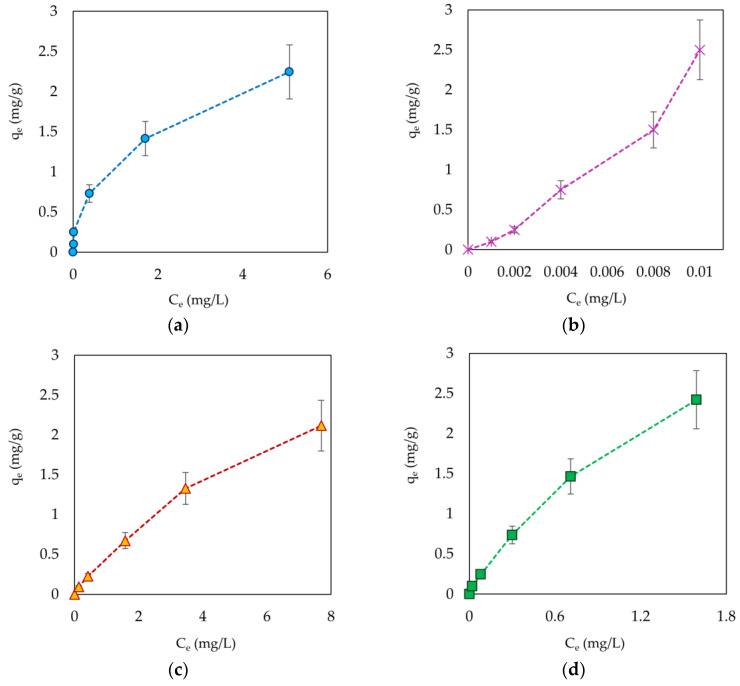
Isothermal dependence between C_e_ and the adsorbents capacity (q_e_): (**a**) CW; (**b**) BChCW; (**c**) WW; (**d**) BChWW.

Figure 6 illustrates the behavior of the adsorbents used for water treatment. The comparison between the equilibrium concentrations and capacity (C_e_ vs. q_e_) provides information on the actual adsorbents efficiency.

**Figure 7:**
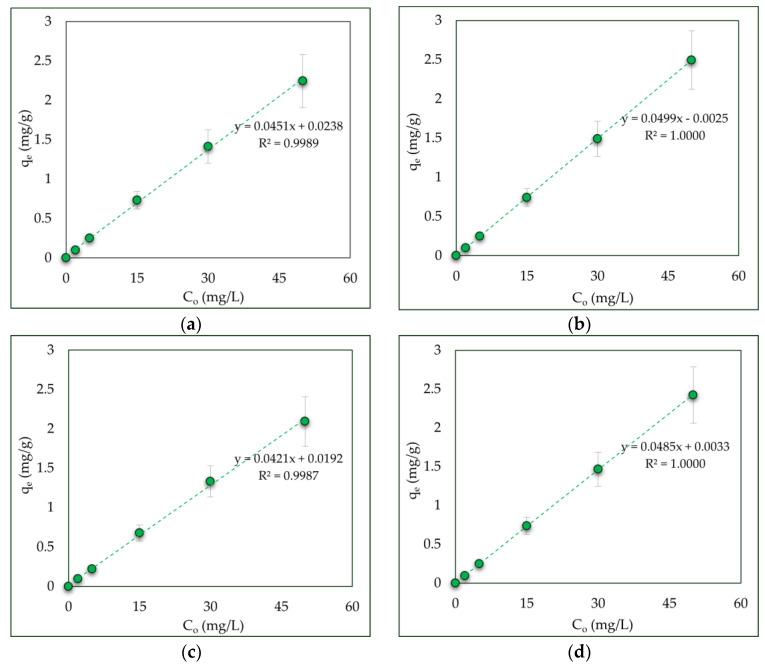
Dependence of adsorption capacity (mean ± SEM, n = 3) on the initial concentration of the pollutant in the solution: (**a**) CW; (**b**) BChCW; (**c**) WW; (**d**) BChWW.

The residual concentration of the pollutants is a critical indicator for the application of adsorbents in real-world water treatment conditions. In this context, the samples BChWW and BChCW show the lowest residual concentrations of the manganese ions, indicating a high affinity for the pollutant. The isothermal curves (C_e_ vs. q_e_) reveal the quantitative capacity of the sorbents. Walnut and cherry wood biochars not only retain more manganese ions, but also manage to accumulate a larger amount of them per unit mass of sorbent, especially at higher concentrations. Experimental findings reveal an inverse relationship between the removal efficiency and the adsorption capacity of the tested adsorbents as the initial metal ion concentration increases, at a constant adsorbent dosage. This behavior suggests that higher contaminant concentrations accelerate the saturation of available adsorption sites, diminishing the adsorbent’s effectiveness and eventually leading to rapid exhaustion. The equilibrium adsorption capacities of the CW, WW, BChCW, and BChWW, achieved at each tested initial concentration of manganese ions, are presented in Figure 7. The adsorption capacity of all adsorbents used increased linearly with increasing initial Mn(II) concentration, which indicates a concentration dependent adsorption process. The exceptionally high correlation coefficients (R^2^ = 0.9989–1.0000) indicate a strong linear relationship, suggesting a potentially uniform adsorption behavior within the studied concentration range, and further experimental results processing using adsorption models reveals the exact mechanism of manganese ion removal from aquatic media at the studied conditions. The observed increase in standard deviation at higher concentrations reflects the natural variability of the adsorption equilibrium at higher metal loadings. It is also probably due to the low number of replicates (n = 3). As a result of the equilibrium studies, the maximum adsorption capacities ranged from 2.12 ± 1.22 to 2.25 ± 1.30 mg/g for WW and CW, and from 2.42 ± 1.44 to 2.50 ± 1.39 mg/g for BChWW and BChCW, respectively. These values were obtained at an initial Mn(II) concentration of 50 mg/L using 1 g of each adsorbent.

**Figure 8:**
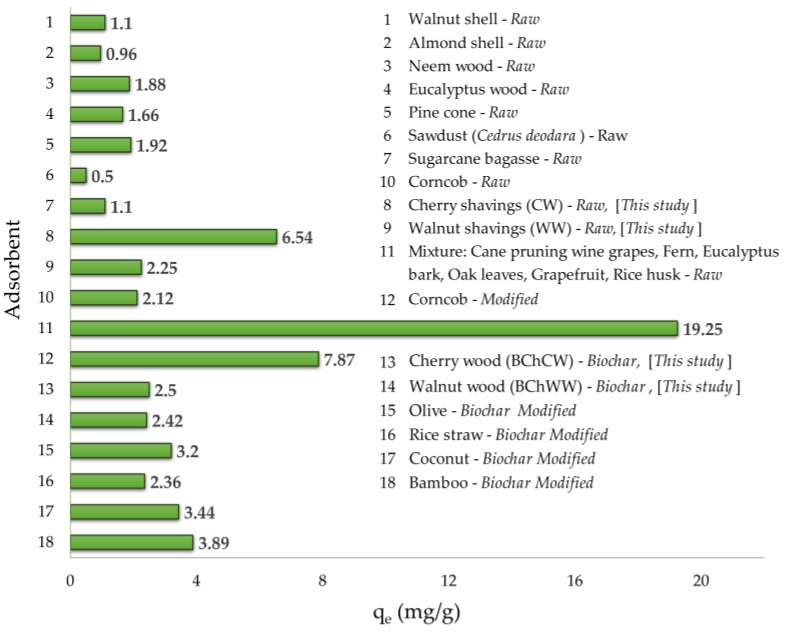
Comparison of the Mn(II) adsorption capacities of CW, WW, BChCW, and BChWW with those of other lignocellulosic biosorbents [52,53,54,55,56].

To the best of our knowledge, no previous studies have reported the use of cherry or walnut wood shavings and their derived biochars for the removal of Mn(II) from aqueous media, which highlights the novelty of the present work. Figure 8 presents a comparative overview of the maximum adsorption capacities obtained in this study against those reported by other researchers using various raw or modified agroforestry materials [52,53,54,55,56]. Although direct analogs are missing, the studies on similar biomass-based sorbents have shown comparable manganese removal efficiencies. This comparison indicates that cherry and walnut wood wastes, along with their biochars, possess promising adsorption potential, most likely due to their high lignin and phenolic content, which are key features known to enhance metal-binding affinity.

All adsorbents tested have high removal efficiencies above 84.62% (Table 4). As shown in the table, the CW is a better adsorbent than WW, and the BChCW is better than BChWW. The removal efficiency of the biochars is higher than that of the raw materials, approaching 100%. Under equilibrium conditions, the BChCW demonstrates the highest efficiency across the studied concentrations range (2–50 mg/L) in comparison with the other adsorbents used. The results are directly related to those obtained from surface characterization of the studied materials.

**Figure 9:**
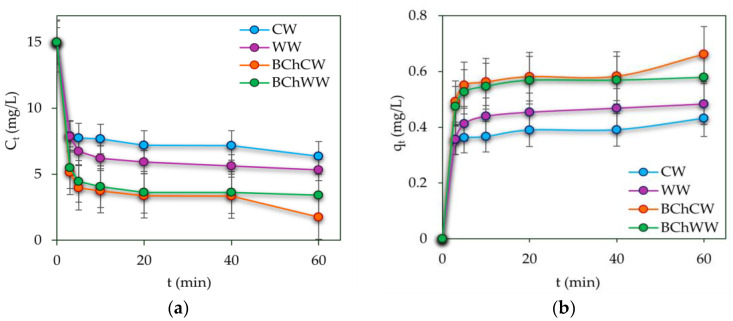
Mn(II) adsorption kinetics at an initial concentration of 15 mg/L using 1 g of each adsorbent: (**a**) Decrease in Mn(II) concentration in aqueous media over time; (**b**) Increase in Mn(II) adsorbed per unit mass of biosorbents over time.

The change in the manganese ions concentration over the time from each adsorbent used is shown on Figure 9.

As a result of the kinetic studies, the maximum adsorption capacities ranged from 0.48 ± 0.28 to 0.43 ± 0.25 mg/g for WW and CW, and from 0.58 ± 0.33 to 0.66 ± 0.38 mg/g for BChWW and BChCW, respectively. These values were obtained at an initial Mn(II) concentration of 15 mg/L, using 1 g of each adsorbent with a contact time of 60 min.

The combined interpretation of equilibrium and kinetic modeling provides a comprehensive understanding of the adsorption behavior of manganese ions onto the studied sorbents—raw CW, WW, and their corresponding biochars (BChCW, BChWW). Each modeling approach highlights distinct aspects of the adsorption process. The isotherm models describe the equilibrium capacity and surface properties, while the kinetic models provide insights into the rate and mechanism of uptake. The experimental equilibrium data were analyzed using the isotherm models of Freundlich, Baudu, and RALF, while the experimental kinetics data were processed using the kinetic models of pseudo second-order, Elovich, and intraparticle diffusion. The isotherm and kinetic model parameters are obtained using nonlinear regression and are presented in Table 5 and Table 6.

Among the tested isotherm models, the Freundlich model obtained the most adequate description of Mn(II) adsorption on cherry wood, with excellent statistical agreement (R^2^ = 0.997, SSE = 0.012, RMSE = 0.055). The parameters K_f_ = 1.127 and 1/n = 0.423 indicate heterogeneous chemisorption, consistent with the presence of reactive oxygen-containing groups in lignocellulosic structures. The Langmuir model, which assumes monolayer adsorption on a homogeneous surface, also showed a good fit (R^2^ = 0.987), although its parameters were less physically consistent. In contrast, the Baudu model yields unrealistic parameters (e. g. negative x, excessively high b_0_), despite high R^2^, confirming that it lacks physical validity. The pseudo second-order model best represents the experimental kinetic results (R^2^ = 0.989, q_e_ = 16.26 mg/g, k_2_ = 0.114 g/mg.min, RMSE = 0.685), indicating that chemisorption controls the adsorption rate. The Elovich model also provides goodness (R^2^ = 0.995, RMSE = 0.451), and confirming surface heterogeneity and energetic diversity. The intraparticle diffusion model (R^2^ = 0.523, RMSE = 4.45) fits poorly, indicating that internal diffusion is not rate-limiting. Based on the data obtained, it could be assumed that the CW removes Mn(II) mainly through chemisorption on oxygenated surface sites, characterized by strong binding energy, fast kinetics, and low capacity, rather than ideal monolayer coverage.

For walnut wood (WW), the Freundlich isotherm model provided a very good equilibrium fit (R^2^ = 0.995, K_f_ = 0.514, 1/n = 0.701, RMSE = 0.073), indicating moderate surface heterogeneity and a mixed mono- and multilayer adsorption process. The Langmuir model fitted the experimental data almost perfect (R^2^ = 0.999), suggesting the possible formation of a near-monolayer at higher Mn(II) loadings. The RALF model (R^2^ = 0.999, RMSE = 0.028) fits perfectly, confirming the heterogeneous monolayer process with realistic parameter values. The kinetic results fit nearly perfectly with the pseudo second-order model (R^2^ = 0.999, q_e_ = 19.36 mg/g, RMSE = 0.261), suggesting that chemisorption governs the rate. The Elovich model (R^2^ = 0.994, RMSE = 0.589) further gives an idea of the heterogeneous adsorption sites with a lower initial sorption rate (λ = 1.63 × 10^4^). The intraparticle diffusion model shows poor fitting (R^2^ = 0.565, RMSE = 4.91) as at CW. WW exhibits efficient chemisorption on moderately heterogeneous surfaces, characterized by slightly higher capacity than CW and very fast adsorption kinetics, with negligible intraparticle diffusion effects [44].

For biochar derived from cherry wood (BChCW), the Langmuir model yielded a high correlation coefficient (R^2^ = 0.999), but the estimated model parameters are negative. These negative values of q_m_ and K_L_ for the BChCW sample lack physical meaning in the context of monolayer adsorption. However, they arise from the mathematical behavior of the nonlinear regression applied to a system that clearly violates the Langmuir model’s assumptions. As confirmed by BET, SEM, and FTIR results, the cherry wood biochar derived by pyrolysis exhibits a highly heterogeneous, microporous carbon surface with reduced oxygenated functionalities. This structure promotes heterogeneous and complexation-driven adsorption of Mn(II) rather than uniform monolayer coverage on identical sites. Under such conditions, the Langmuir model fitting often yields mathematically optimal but physically unrealistic negative constants [57,58,59,60,61]. The results obtained using the model are not suitable for describing Mn(II) adsorption on BChCW. In contrast, the Freundlich model provides the best equilibrium fit of the experimental data, which were obtained by biochar of cherry wood (R^2^ = 0.984, K_f_ = 1979.986, 1/n = 1.460, RMSE = 0.140). The very high K_f_ and 1/n > 1 values indicate cooperative multilayer adsorption dominated by physicochemical interactions within the pores. The good fit of the RALF model with data (R^2^ = 0.982, RMSE = 0.169) supports the idea of the presence of complex surface heterogeneity and multiple adsorption layers. The kinetic results are best described by the Elovich model (R^2^ = 0.988, RMSE = 0.962), which accounts for the heterogeneous energy distribution of biochar surfaces. The PSO model (R^2^ = 0.956, RMSE = 1.822) also shows reasonable fitting but with higher errors, indicating that the adsorption is partially controlled by intraparticle diffusion (R^2^ = 0.591). Probably the BChCW adsorbs Mn(II) through a cooperative mechanism—initial chemisorption on functional groups, followed by multilayer physisorption in pores. Although the kinetic is slower, and the adsorption capacity increases by several orders of magnitude compared with the raw CW.

The Freundlich isotherm provides a good fit to the data obtained by BChWW (R^2^ = 0.980, K_f_ = 1.768, 1/n = 0.703, RMSE = 0.164), indicating heterogeneous chemisorption with some multilayer contribution. Interestingly, the Langmuir model provided an excellent fit (R^2^ = 1.000) for the BChWW) yielding physically consistent, positive parameters in contrast with the negative results obtained for BChCW. The Langmuir model also fitted the data very well (R^2^ = 1.000), suggesting that the biochar surface may approach monolayer coverage under certain conditions. The intrinsic differences in surface chemistry and porosity arising from the precursor composition could account for this discrepancy. The BChWW retains oxygenated surface functionalities and a relatively homogeneous micro-mesoporous structure, both of which favor monolayer adsorption. In contrast, the aromatic, microheterogeneous surface of BChCW leads to complex, nonuniform Mn(II) binding that violates the Langmuir assumptions. Nevertheless, the RALF model gives the best data fit (R^2^ = 1.000, RMSE = 0.019) and further confirms the consistency of equilibrium data and the hybrid nature of adsorption. Kinetic results show outstanding conformity with the pseudo second-order model (R^2^ = 0.996, q_e_ = 23.92 mg/g, RMSE = 0.478), confirming chemisorption as the main mechanism. The Elovich model (R^2^ = 0.996, RMSE = 0.485) suggests a very high initial adsorption rate (λ = 2.36 × 10^7^), consistent with a highly porous and reactive surface. The intraparticle diffusion model again gives poor correlation (R^2^ = 0.412, RMSE = 5.68), suggesting that internal diffusion is not the dominant mechanism. Based on the data, it could be considered that the BChWW combines high adsorption capacity, fast kinetics, heterogeneous surface structure conducive to efficient Mn(II) chemisorption.

Combined equilibrium and kinetic modeling highlights a dual physicochemical adsorption mechanism, which is predominantly driven by fast surface chemisorption. High coefficients of determination (R^2^ ≈ 1.000) and minimal statistical errors confirm the robustness and reliability of the applied models.

Building on the initial findings of this study, future work will focus on optimizing key operational parameters influencing adsorption performance, particularly the pH at the point of zero charge (pHpzc), which plays a central role in controlling sorbent surface interactions in aqueous environments [62,63,64,65,66]. The competitive adsorption phenomena, especially in multicomponent systems, which have been shown to exhibit complex behavior that significantly affects selectivity and uptake capacities [67,68,69], will also be examined. Additional experiments will explore a broader range of Mn(II) concentrations and the addition of other metal ions, such as Cu^2+^, Fe^2+/3+^, and Zn^2+^, to simulate competitive interactions observed in real wastewaters [70,71,72]. A comprehensive surface and structural characterization of the adsorbents before and after the experiments using FTIR, SEM–EDS, BET, and XPS analyses will be performed to define the adsorption mechanism related to their surface characteristics. Furthermore, the regeneration and reusability of the biosorbents will be examined through multiple adsorption–desorption cycles to evaluate their practical applicability [73]. In cases where regeneration proves infeasible, appropriate strategies for environmentally sound disposal or valorization of the spent materials will be considered, such as safe incineration, landfilling with stabilization, or repurposing in secondary applications, in line with sustainable management practices reported in the literature [74,75].

The outcomes of this research are expected to contribute to the development of sustainable, low-cost, and circular-economy-oriented technologies for the treatment of metal-contaminated wastewaters, with particular relevance to the mining and metallurgical sectors.

## 4. Conclusions

This study investigated the adsorption performance of four adsorbents—raw walnut and cherry wood shavings and their biochars produced via pyrolysis at 700 °C. FTIR characterization confirmed the structural stabilization and aromatization of the wood-derived biochars during pyrolysis, explaining their enhanced adsorption performance. The biochars demonstrated higher adsorption capacities regarding the manganese ions in comparison with the untreated raw materials, probably due to their increased surface area and well-developed pore structures resulting from thermal activation. The experimental data were well described by the applied nonlinear isotherm and kinetic models, as evidenced by high coefficients of determination (R^2^ > 0.95) and low associated statistical errors. The Freundlich model best describes the experimental data for the raw adsorbents, confirming heterogeneous and partially multilayer adsorption. The pseudo second-order and Elovich kinetic models suggest that chemisorption is the dominant rate-controlling mechanism, probably mediated by oxygen functional groups such as hydroxyl, carbonyl, and carboxyl. The regeneration efficiency of these adsorbents, which is an essential factor for their practical applicability, was not evaluated in the present study. On the one hand, these findings confirm that walnut and cherry wood-processing residues can serve as sustainable and low-cost adsorbents for polishing contaminated wastewater with low manganese concentrations, and on the other hand, they could serve as precursors for bio-based adsorbents applicable in water treatment. Future research will aim to optimize operational parameters, explore alternative surface modification strategies, test regeneration, and assess the adsorbents’ performance using multicomponent and real wastewater samples.

## Figures and Tables

**Table 1:** Nonlinear forms of isotherms and kinetics models.

Isotherms Models		Plot
Langmuir	$q_{e}=\frac{q_{m}K_{L}C_{e}}{1+K_{L}C_{e}}$	*q_e_* vs. *C_e_*
Freundlich	$q_{e}=K_{F}{C_{e}}^{n_{F}}$	
Baudu	$q_{e}=\frac{Q_{m_{0}}b_{0}{C_{e}}^{\left(1+x+y\right)}}{1+b_{0}{C_{e}}^{\left(1-x\right)}}$	
RALF	$q_{e}=q_{\max}(1-e^{-\alpha \;C_{e}^{b}})$	
**Kinetic models**		
Pseudo second-order	$q_{t}=\frac{q_{e}^{2}k_{2}t}{1+q_{e}k_{2}t}$	*q_t_* vs. *t*
Elovich	$q_{t}=\frac{1}{\beta }\ln(1+\alpha \beta t)$	
Intraparticle diffusion model	$q_{t}=k_{id}t^{0.5}+C$	*q_t_* vs. *t*^1/2^

**Table 2:** Chemical composition.

Industrial Wood Processing Residues	Moisture Content (%)	Lignin (%)	Cellulose (%)	Ash (%)	Substances Soluble in Hot Water (%)	Extractables (Ethanol-Benzene) (%)
CW	12.52	18.28	50.31	1.04	0.62	1.04
WW	13.06	20.84	47.15	2.17	3.21	2.24

**Table 3:** Surface characteristics of studied materials (mean ± SEM, n = 3).

Adsorbent	Surface Area (m^2^/g)	Pore Volume (cm^3^/g)	Pore Width (nm)
CW	2.321 ± 0.15	0.003 ± 0.007	2.313 ± 0.26
WW	1.057 ± 0.21	0.002 ± 0.01	2.647 ± 0.33
BChCW	512.8 ± 6.29	0.269 ± 0.01	1.126 ± 0.10
BChWW	396.0 ± 8.42	0.269 ± 0.02	1.178 ± 0.13

**Table 4:** Removal efficiency of green adsorbents and their biochars at the studied manganese concentration range.

C_0_ (mg/L)	RE (%)			
Mn(II)	CW	WW	BChCW	BChWW
0.00	0.00	0.00	0.00	0.00
2.00	98.50	93.50	99.95	99.00
5.00	97.80	91.60	99.96	98.40
15.00	97.47	89.07	99.97	98.00
30.00	94.33	88.47	99.97	97.63
50.00	89.80	84.62	99.98	96.82

**Table 5:** Values of the equilibrium model parameters and error functions for the systems studied.

Model	Parameter	CW	WW	BChCW	BChWW	Error	CW	WW	BChCW	BChWW
Langmuir	q_m_	2.766	4.380	−2.255	5.065	R^2^	0.987	0.999	0.990	1.000
	K_L_	0.742	0.122	−52.161	0.575	SSE	0.094	0.003	0.040	0.002
						MSE	0.031	0.001	0.013	0.001
						RMSE	0.177	0.031	0.115	0.028
Freundlich	K_f_	1.127	0.515	1450.000	1.768	R^2^	0.996	0.995	0.981	0.980
	1/n	0.423	0.701	1.395	0.703	SSE	0.012	0.016	0.079	0.081
						MSE	0.004	0.005	0.026	0.027
						RMSE	0.063	0.073	0.162	0.164
RALF	q_max_	13.519	2.906	48.655	3.893	R^2^	0.997	0.999	0.982	1.000
	a	0.088	0.177	27.634	0.639	SSE	0.013	0.002	0.086	0.001
	b	0.445	0.979	1.374	0.906	MSE	0.004	0.001	0.029	0.000
						RMSE	0.065	0.028	0.169	0.019
Baudu	q_m0_	1.131	2.309	872.076	2.626	R^2^	0.996	0.999	0.965	0.999
	b_0_	2233.492	0.255	3.296	2.215	SSE	0.016	0.004	0.225	0.003
	x	−0.019	0.042	0.321	0.337	MSE	0.005	0.002	0.075	0.001
	y	0.458	0.091	0.192	−0.225	RMSE	0.074	0.045	0.274	0.037

**Table 6:** Values of the kinetics model parameters and error functions for the systems studied.

Model	Parameter	CW	WW	BChCW	BChWW	Error	CW	WW	BChCW	BChWW
Pseudo second-order model	q_e_	16.261	19.357	27.423	23.920	R^2^	0.989	0.999	0.956	0.996
	k_2_ (g/mg·min)	0.114	0.052	0.026	0.054	SSE	2.347	0.341	29.862	2.057
						MSE	0.469	0.068	3.318	0.229
						RMSE	0.685	0.261	1.822	0.478
Elovich	β (g/mg)	1.132	0.686	0.450	0.898	R^2^	0.995	0.994	0.988	0.996
	λ (mg/g⋅min)	2.14 × 10^6^	1.63 × 10^4^	5.32 × 10^3^	2.36 × 10^7^	SSE	1.017	1.737	8.334	2.113
						MSE	0.203	0.347	0.926	0.235
						RMSE	0.451	0.589	0.962	0.485
Intraparticle diffusion model	k_id_ (mg/g⋅min^0.5^)	1.573	1.890	1.382	0.986	R^2^	0.523	0.565	0.591	0.412
	C (mg/g)	7.358	8.012	13.832	14.209	SSE	99.037	120.332	277.228	290.870
						MSE	19.807	24.066	30.803	32.319
						RMSE	4.451	4.906	5.550	5.685

## Data Availability

The original contributions presented in the study are included in the article. Further inquiries can be directed to the corresponding author.
